# Computational estimates of annular diameter reveal genetic determinants of mitral valve function and disease

**DOI:** 10.1172/jci.insight.146580

**Published:** 2022-02-08

**Authors:** Mengyao Yu, Catherine Tcheandjieu, Adrien Georges, Ke Xiao, Helio Tejeda, Christian Dina, Thierry Le Tourneau, Madalina Fiterau, Renae Judy, Noah L. Tsao, Dulguun Amgalan, Chad J. Munger, Jesse M. Engreitz, Scott M. Damrauer, Nabila Bouatia-Naji, James R. Priest

**Affiliations:** 1 Department of Pediatrics and; 2 Division of Cardiovascular Medicine, Stanford University School of Medicine, Stanford, California, USA.; 3 Paris Cardiovascular Research Center, INSERM, University of Paris, Paris, France.; 4 College of Information & Computer Sciences at University of Massachusetts Amherst, Amherst, Massachusetts, USA.; 5 University of Nantes, INSERM, CNRS, CHU Nantes, The Thorax Institute, Nantes, France.; 6 Department of Surgery, University of Pennsylvania, Philadelphia, Pennsylvania, USA.; 7 Department of Genetics, Stanford University School of Medicine, Stanford, California, USA.; 8 Basic Science & Engineering Initiative & Betty Irene Moore Children’s Heart Center, Lucile Packard Children’s Hospital, Stanford, California, USA.; 9 Chan-Zuckerberg Biohub, San Francisco, California, USA.

**Keywords:** Cardiology, Genetics, Cardiovascular disease, Diagnostic imaging, Population genetics

## Abstract

The fibrous annulus of the mitral valve plays an important role in valvular function and cardiac physiology, while normal variation in the size of cardiovascular anatomy may share a genetic link with common and rare disease. We derived automated estimates of mitral valve annular diameter in the 4-chamber view from 32,220 MRI images from the UK Biobank at ventricular systole and diastole as the basis for GWAS. Mitral annular dimensions corresponded to previously described anatomical norms, and GWAS inclusive of 4 population strata identified 10 loci, including possibly novel loci (*GOSR2*, *ERBB4*, *MCTP2*, *MCPH1*) and genes related to cardiac contractility (*BAG3*, *TTN*, *RBFOX1*). ATAC-Seq of primary mitral valve tissue localized multiple variants to regions of open chromatin in biologically relevant cell types and rs17608766 to an algorithmically predicted enhancer element in *GOSR2*. We observed strong genetic correlation with measures of contractility and mitral valve disease and clinical correlations with heart failure, cerebrovascular disease, and ventricular arrhythmias. Polygenic scoring of mitral valve annular diameter in systole was predictive of risk mitral valve prolapse across 4 cohorts. In summary, genetic and clinical studies of mitral valve annular diameter revealed genetic determinants of mitral valve biology, while highlighting clinical associations. Polygenic determinants of mitral valve annular diameter may represent an independent risk factor for mitral prolapse. Overall, computationally estimated phenotypes derived at scale from medical imaging represent an important substrate for genetic discovery and clinical risk prediction.

## Graphical abstract

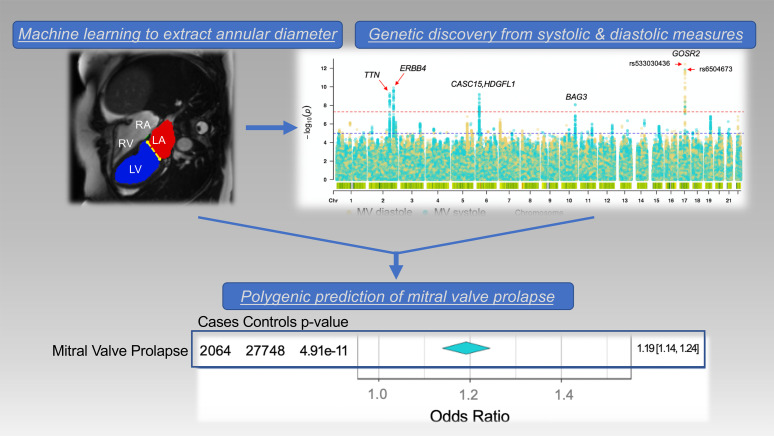

## Introduction

The mitral valve is a 2-leaflet structure anchored to the annulus with secondary chordal attachments to the ventricle, which facilitates blood flow from the left atrium to the ventricle and prevents regurgitant flow into the left atrium during ventricular systole. The annulus of the mitral valve is an ellipsoidal D-shaped fibrous ring, and the anterior-posterior diameter may be measured clinically in the 4-chamber view of cardiac imaging by echocardiogram, computed tomography, or MRI (1).

Both the annulus and leaflets of the mitral valve arise from the atrioventricular cushions during early cardiac development during weeks 5–6 after fertilization in human cardiovascular development (2, 3).The normal process of valvulogenesis may be disturbed in Mendelian forms of disease or, alternately, acquired pathology localized to the leaflets or the annulus may compromise mitral valve function, resulting in either regurgitation or stenosis (4). In mitral valve prolapse (MVP) one or both leaflets of the mitral valve may become thickened or dysplastic and deviate into the left atrium during ventricular systole typically causing regurgitation, and risk for MVP is influenced by the size of the mitral annulus (5). Recent work has described the role of common genetic variation as affecting cellular alignment, ciliary function, and TGF-β signaling in MVP (6, 7).

Recent work has linked the genetics governing normal variation in the size of cardiovascular structures derived from medical imaging of the heart to insights into cardiovascular biology as well as common and rare disease (8, 9). Here, we report automated extraction of mitral valve annular diameter at systole and diastole from cardiac MRI data. Extracted data corresponded to previously described anatomical norms, and GWAS inclusive of 4 population substrata identified 10 loci, including loci that have potentially not been described before and loci previously implicated in mitral valve biology or cardiac contractility. Polygenic scoring of the mitral valve annular diameter was predictive of mitral valve regurgitation across 4 cohorts.

## Results

**Figure 1:**
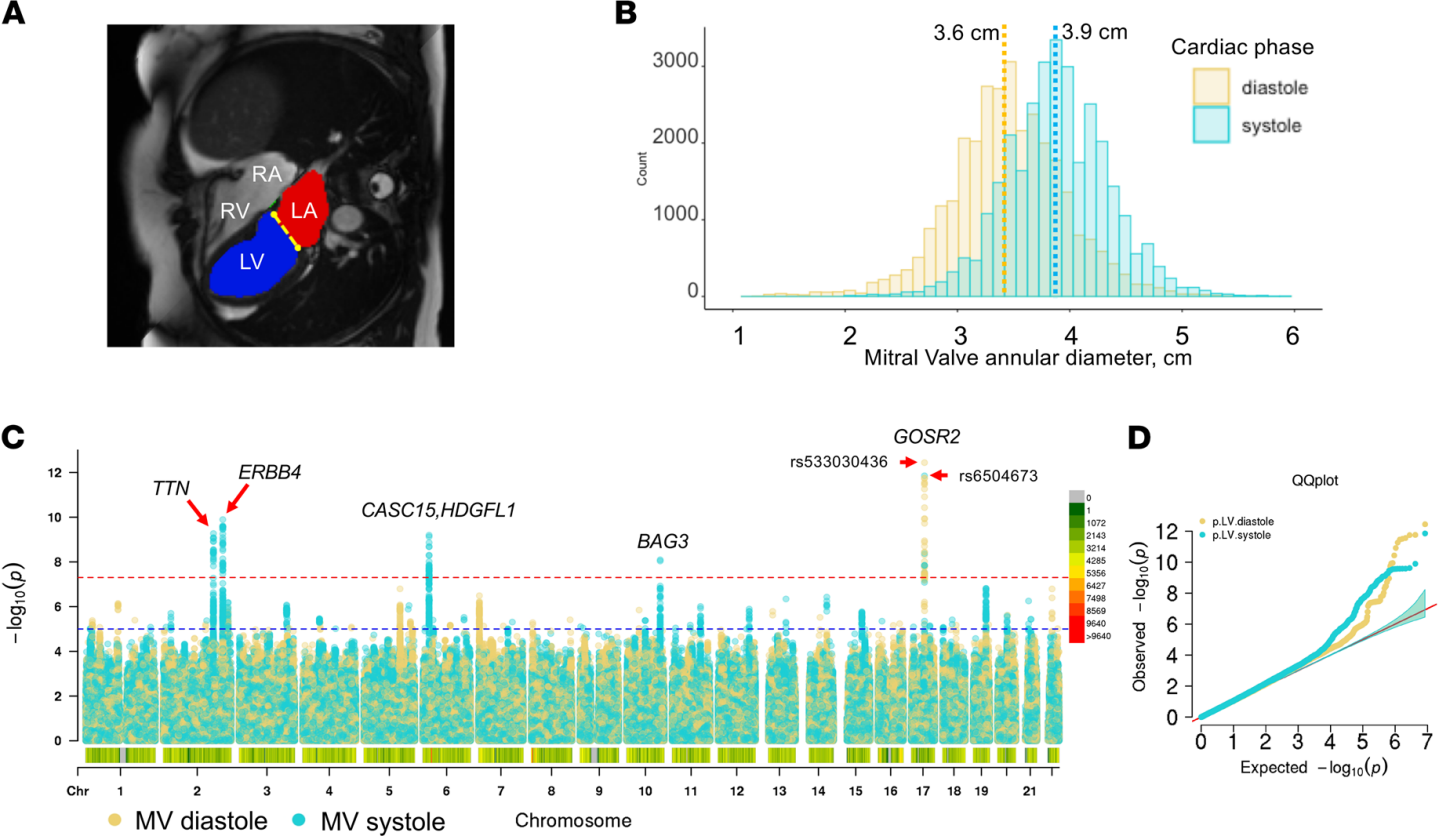
Genome-wide association of automated measures of mitral valve annulus diameter. (**A**) Still frame of a cardiac MRI in the 4-chamber view demonstrating segmentation of the left ventricle (LV; indicated by the blue region), left atrium (LA; indicated by the red region), and the diameter of the annulus of the mitral valve in the anterior-posterior view (indicated by the yellow dotted line). (**B**) Population distribution of mitral valve annular diameter estimates in ventricular systole and diastole, which conform to previous population-based estimates derived from cardiac MRI. As expected, systolic measurements are greater than diastolic measurements, as the annulus experiences deformation during ventricular contraction. Mean values are indicated by dotted lines and labeled. (**C**) Manhattan plot of individuals of European ancestry, highlighting unique and shared loci for MV annulus diameter in systole (light blue) and diastole (yellow) (**D**) Quantile-quantile plot indicates an absence of systematic inflation of genetic determinants for both measurements.

After exclusion of outliers and individuals ± 2.5 standard deviation, estimates of mitral valve annular diameter were available at 2 different time points during the cardiac cycle (31,973 left ventricle [LV] end-systole, 31,864 LV end-diastole) that conformed to previously described population-based norms (10) (Figure 1, A and B). Automated measures were on average 0.7 mm or 2.3% larger than manual measures with no systematic relationship of the error in mitral valve annular measurement to body surface area, genetic sex, age, or imaging acquisition error observed across 100 randomly selected test images. We performed the main GWAS on mitral valve annular diameter measured at LV systole (*n* = 27,156) and LV diastole (*n* = 27,074), followed by the replication GWAS, where we observed genetic signals meeting standard levels of threshold for genome-wide significance for both Mitral valve annular diameter was measured in diastole and systole (Figure 1, C and D). Results from systolic measurements centered around known loci related to cardiac contractility (*TTN*, chr2, rs80182096, *P*_global_ = 5.37 × 10^-10^ and *BAG3*, chr10, rs17099139, *P*_global_ = 8.02 × 10^-9^) as well as novel loci (*ERBB4*, chr2, rs4673661, *P*_global_ = 1.28 × 10^-10^ and an intergenic locus at 6p22.3 proximal to *HDGFL1*, chr6, rs10485012, *P*_global_ = 6.33 × 10^-10^), while a set of intronic variants in *GOSR2* at chr17 displayed different lead variants identified in systole (rs6504673, *P*_global_ = 1.40 × 10^-12^) and diastole (rs533030436, *P*_global_ = 3.63 × 10^-13^), which are unlinked (R^2^ = 0.02) within European populations (Table 1 and Supplemental Figure 1; supplemental material available online with this article; https://doi.org/10.1172/jci.insight.146580DS1). We also performed an analysis of rare variants of high imputation quality (minor allele frequency [MAF] < 0.01), revealing 3 noncoding variants; the first closely localized to *NUBL* for mitral valve annular diameter measured in diastole, and additional variants in *SLC35F6* and *ST7* showed an association for annular diameter in systole (Supplemental Table 1). A gene-based analyses using eMAGMA (v1.08) examined a multiplicity of evidence for assignment of a causal gene and tissue-specific localization for which the variant rs80182096 was localized to *TTN* by 3D chromatin interactions in mesendoderm (*P*_FDR_ = 5.3× 10^–60^) and LV (*P*_FDR_ = 2.1× 10^–41^). The other identified loci were not clearly localized by expressed quantitative trait loci (eQTL) data or open chromatin to a single myocardial or vascular tissue lineage included in the available databases but instead relied upon proximal genes within the locus (Supplemental Figure 2).

**Figure 2:**
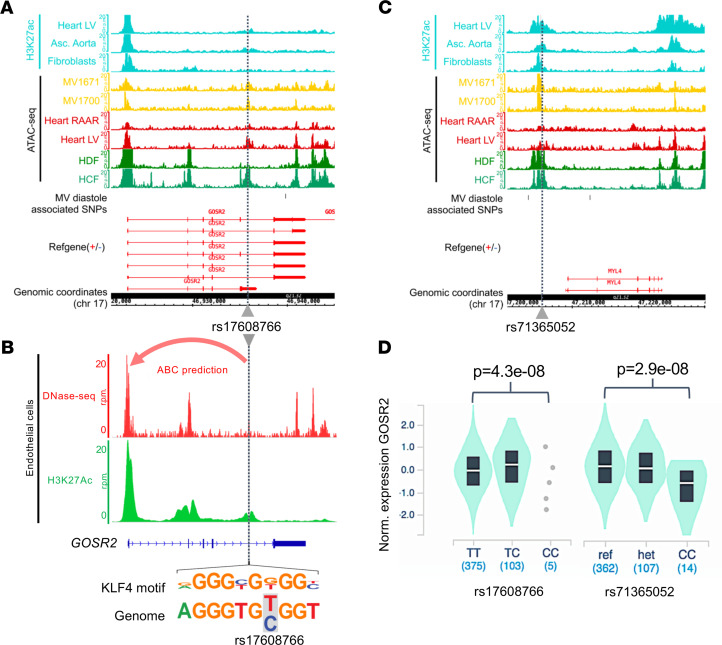
Variation associated with mitral valve annular diameter in systole and diastole is centered in open chromatin regions around *GOSR2*. (**A**) Visualization of ATAC-Seq/histone ChIP read densities (in reads/million, r.p.m.) at chromosome 17 locus in the regions surrounding rs17608766, which is associated with mitral valve annular diameter in diastole. Notably, the region of open chromatin is present in ATAC-Seq data derived from primary mitral valve tissue (MV1671 and MV1700). (**B**) The same variant rs17608766 overlaps open chromatin regions present in cardiovascular precursors and endothelial cells but not embryonic stem cells (hESCs) and occurs within an activity-by-contract (ABC) enhancer element predicted to interact with the promoter of *GOSR2* in heart ventricle tissue and endothelial cells (red arrow) and specifically disrupts a KLF4 binding motif. (**C**) ATAC-Seq/histone ChIP read densities for the locus of rs71365052 associated with mitral valve annular diameter in systole, which appears in a large region of open chromatin proximal to *MYL4*. (**D**) Violin plot representation of association between genotype and GOSR2 expression in cultured fibroblasts for rs17608766 and rs71365052 derived from GTEx (v8 release) shows strong relationship with GOSR2 for both variants in opposite directions. There is a notable absence of linkage between the two variants (*r*^2^ = 0.03). Both risk alleles (rs1768766-C and rs71365052-CTG) are associated with higher expression of GOSR2 and larger mitral annular diameter. MV, normal valves; HDF, human dermal fibroblasts; HCF, human cardiac fibroblasts; Heart LV, heart left ventricle; Heart RAAR, heart right atrium auricular region; Asc. Aorta, ascending aorta; HMVEC, human microvascular endothelial cells; HUVEC, human umbilical endothelial cells.

Given that mitral valve annular tissue is not specifically represented in eQTL data from GTEx and has been absent from data sets of chromatin structure, we sought to look for specific overlap of identified loci with chromatin confirmation in samples of mitral valve tissue and more detailed developmental assays in silico. We examined variants overlapping open chromatin regions from mitral valve tissue and a variety of other cell types among replicated lead variants and variants in high linkage disequilibrium (LD) (*r*^2^ ≥ 0.5) in European populations. We found overlapping variants at 3 of the 6 loci (Supplemental Table 2). At the chromosome 17 locus rs1768766 is in strong linkage with mitral valve diastolic diameter associated variant (rs533030436) and overlaps open chromatin in primary mitral valve tissue and fibroblasts (Figure 2A), resulting in a disruption of a canonical KLF4 binding motif within an activity-by-contact predicted endothelial cell enhancer interacting with the promoter of *GOSR2* (Figure 2B). At the same locus, an insertion variant from a different haplotype (Supplemental Figure 9), rs71365052, is in linkage with mitral valve systolic diameter associated variant rs6504673 and overlaps a prominent open chromatin region in mitral valve and fibroblasts, as well as strong active enhancer marks in heart, fibroblasts, and aorta (Figure 2C). Interestingly, both variants appear to be eQTLs of GOSR2 transcripts in fibroblasts (Supplemental Table 2 and Figure 2D), even though rs71365052 is located more than 200 kb downstream of the *GOSR2* locus. At the chromosome 2 locus proximal to *CCDC141*, one variant (rs60105920), in strong LD with lead SNP rs10485012, overlapped a prominent open chromatin region in valve tissue and fibroblasts, as well as active enhancer histone marks in heart tissue and aorta (Supplemental Figure 3A). This variant is an eQTL for *FKBP7* in primary fibroblasts, located more than 300 kb upstream of the top associated variants (Supplemental Figure 3B).

We also performed a trans-ancestry meta-analysis (Supplemental Figure 4), which revealed loci meeting genome-wide significance for mitral valve annulus diameter at diastole (*MCTP2*, *MCPH1*) and systole (*MROH7*, and an intergenic locus at 16q21), with lead variants discovered among the African/Afro-Caribbean population strata (Supplemental Figure 5). Candidate signals of interest were observed in mitral valve measurements from the 2 smaller population strata of South and East Asian descent but did not meet standard significance thresholds in the trans-ancestry meta-analysis (Supplemental Table 3).

**Figure 3:**
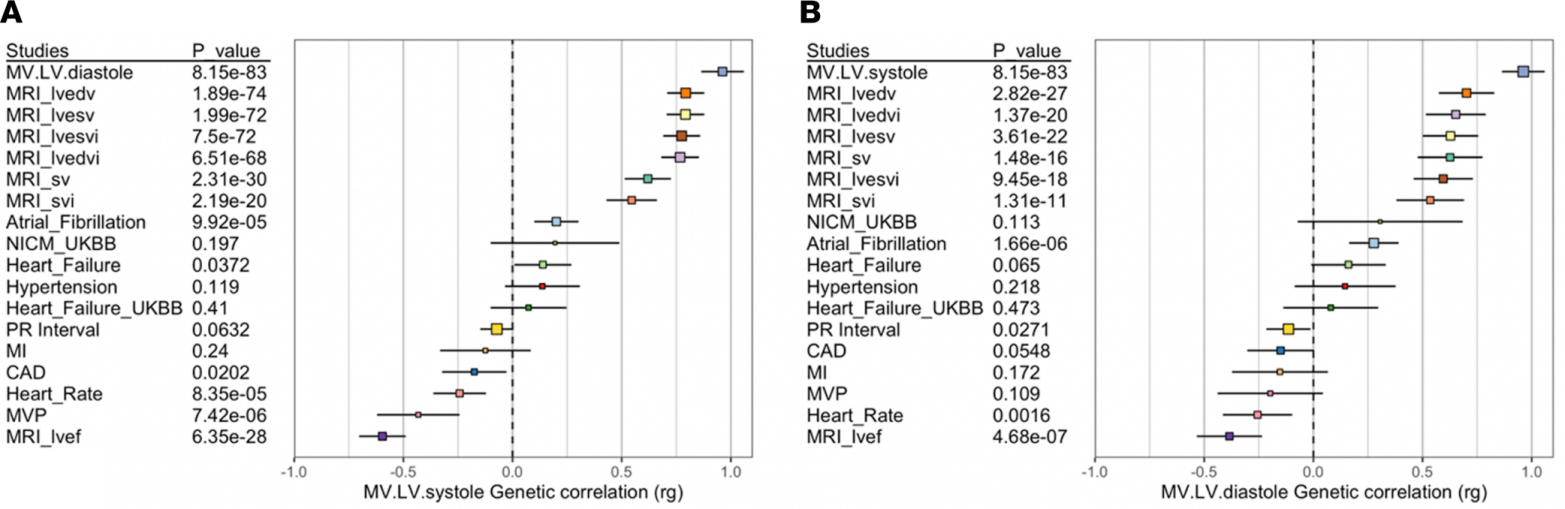
Forest plots showing genetic correlation of mitral valve annular diameter with related cardiovascular phenotypes. (**A**) For annular diameter measured at systole there is a strong positive correlation with indexed measures of left ventricular volume, obtained from a largely overlapping data set as well as with atrial fibrillation, and a negative correlation with heart rate, mitral valve prolapse, and ejection fraction. (**B**) Annular diameter measured at diastole displays many of the same correlations, as well as a positive correlation with atrial fibrillation. lv, left ventricular; edv, end diastolic volume; esv, end systolic volume; sv, stroke volume; i, indexed to body surface area; ef, ejection fraction; NICM, nonischemic cardiomyopathy; MI, myocardial infarction; CAD, coronary artery disease; MVP, mitral valve prolapse.

Genome-wide significant variants for systole were each nominally significant (*P* < 0.05) for diastole and vice versa (Supplemental Table 4), and LD score correlation (LDSC) confirmed a substantial genetic overlap in the genetic basis of the 2 measurements of mitral annulus diameter, as would be expected in measures derived from the same tissue in the same set of individuals (*P* = 8.15 × 10^–83^) (Figure 3). In addition, for measures of mitral annulus diameter at systole and diastole, LDSC suggested a strong positive genetic correlation with GWAS of LV volumes, stroke volume, and ejection fraction derived from analyses of a largely overlapping data set (8) and a strong negative correlation with ejection fraction and heart rate (Figure 3). Of note, MVP displayed a negative genetic correlation with mitral valve annulus measurement at systole (*P* = 7.42× 10^–6^), while atrial fibrillation displayed a positive genetic correlation with annulus measurement at diastole (*P* = 1.66× 10^–6^).

**Figure 4:**
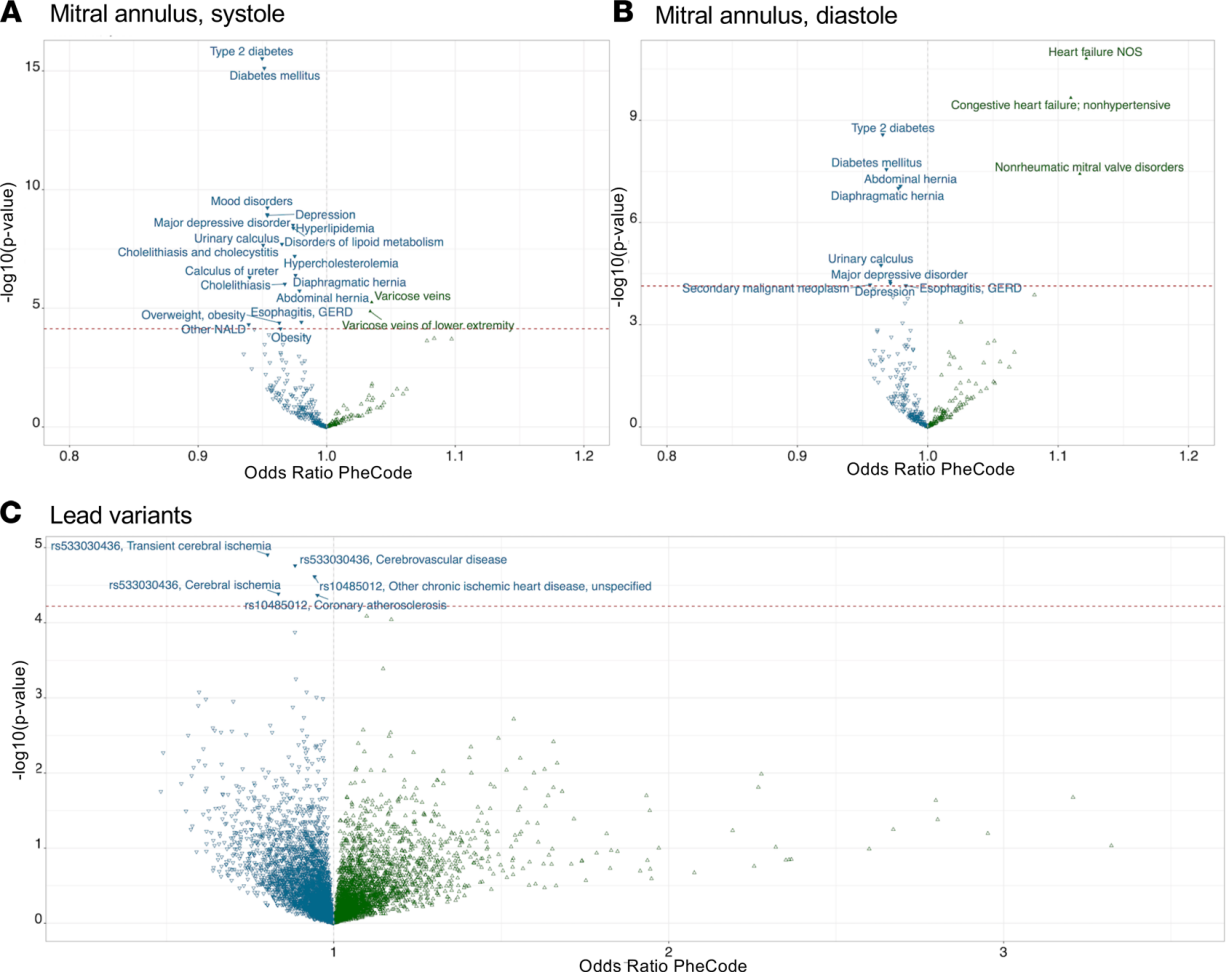
PheWAS of estimated measures of mitral annulus size and variants identified in GWAS identifies clinical correlates of disease. (**A**) PheWAS of mitral annular diameter in systole among 32,690 individuals with a measurement. The *x* axis represents the odds ratio for a PheCODE, and the *y* axis represents the negative logarithm of the *P* value of the association between a PheCODE and the measurement. Larger annular diameter measured in systole is positively associated with varicose veins and negatively associated with a variety of cardiometabolic traits most notably diabetes (OR = 0.95, *P* = 3.02 × 10^–16^). (**B**) PheWAS of mitral annular diameter in diastole among 32,093 individuals with a measurement. Larger annular diameter measured in diastole is positively associated with nonrheumatic mitral valve disorders (OR = 1.12, *P* = 3.74 × 10^–8^) and heart failure (OR = 1.12, *P* = 1.56 × 10^–11^) and negatively associated with cardiometabolic traits and abdominal and diaphragmatic hernias. (**C**) A PheWAS of 5 lead variants identified in the GWAS within individuals without a mitral valve measurement (*n* = 308,683), suggesting that the C allele of rs10485012 (associated with larger annular diameter in systole) was associated with decreased risk of coronary atherosclerosis (OR = 0.95, *P* = 4.29 × 10^–5^) and heart failure (OR = 0.92, *P* = 8.4 × 10^–4^), while the G allele of rs533030436 (associated with larger annular diameter in diastole) specifically displayed an association with decreased risk for cerebrovascular disease (OR = 0.88, *P* = 1.73 × 10^–5^) and related phenotypes.

To better understand the clinical correlates of our anatomical estimates and genetic findings, we performed phenome-wide association studies (PheWAS) of mitral annular diameter among the individuals with a measurement. Among the 32,219 individuals with a measurement, a larger annular diameter in diastole and systole were positively associated with heart failure and cardiomyopathy, while smaller annular diameter in both measures was associated with type 2 diabetes, hyperlipidemia, and hernias affecting the diaphragmatic surface of the heart (Figure 4, A and B). We also performed a PheWAS of 4 lead variants identified in the GWAS within individuals without a mitral valve measurement (*n* = 308,683); it suggested that the C allele of rs10485012 (associated with larger annular diameter in systole) was associated with decreased risk of coronary atherosclerosis and heart failure, while the G allele of rs533030436 (associated with larger annular diameter in diastole) specifically displayed an association with decreased risk for cerebrovascular disease (Figure 4C).

**Figure 5:**
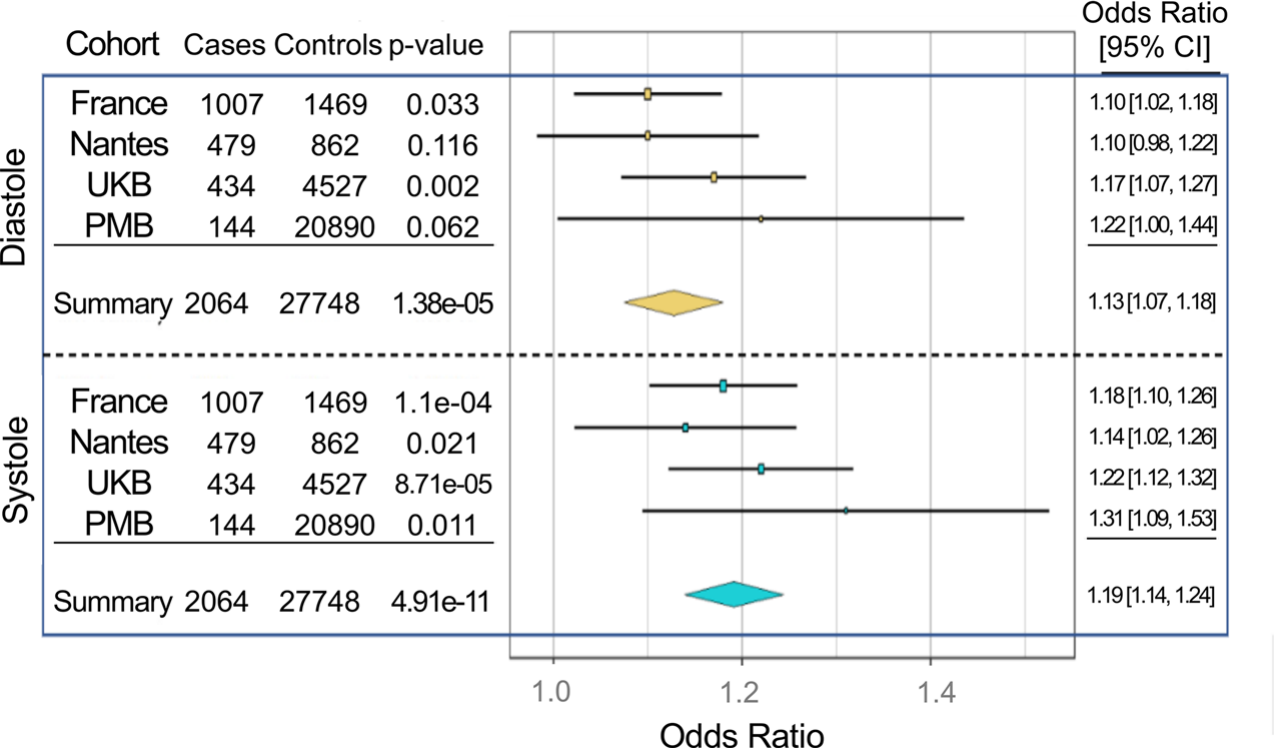
Forest plots relating the polygenic risk score for mitral valve annular diameter measured in systole and diastole to the risk of mitral valve prolapse across 4 independent cohorts. OR measured per standard deviation increase in polygenic risk score.

Interestingly, the PheWAS for mitral valve annular diameter in diastole specifically highlighted the direct association with nonrheumatic disorders of the mitral valve (Figure 4A). Therefore, we created a polygenic score for mitral valve annular diameter for the diastolic and systolic measurements using the validation data set (described above) subdivided into 2 groups that captured a larger amount of variation for the systolic measurement (*r*^2^ = 0.011, *P* = 6.7 × 10^–11^) than the diastolic measurement (*r*^2^ = 0.006, *P* = 3.1 × 10^–7^) (Supplemental Figure 6 and Supplemental Table 5). Across 4 separate cohorts of MVP, the polygenic score from both measures behaved as expected, with the mitral annular score from systole capturing a larger proportion of *r*^2^ than the diastolic measure. The polygenic score for mitral valve annular diameter measured at ventricular systole displayed the strongest prediction of risk for MVP or regurgitation ranging from an OR of 1.14–1.31 between the different cohorts (Figure 5). In meta-analysis across the 4 cohorts, per standard deviation increase in polygenic score of mitral valve annular diameter at systole, there was a 1.1-fold increase in the risk of MVP (OR = 1.19; 95% CI, 1.14–1.24; *P* = 4.9 × 10^–11^), while the risk related to mitral valve annular diameter at diastole was lower (OR = 1.13; 95% CI, 1.07–1.18; *P* = 1.38 × 10^–5^) (Figure 5). For each standard deviation increase in the polygenic score for mitral annular diameter at systole we observed a small increased risk of mitral valve regurgitation in the Penn Medicine BioBank that did not meet nominal thresholds for statistical significance (Supplemental Figure 7). Finally, using the polygenic scores generated we also performed a PheWAS of individuals with samples at the UK Biobank without imaging data (*n* = 308,683). The polygenic risk score (PRS) for mitral valve annular diameter at diastole was positively associated with varicose veins and ventricular tachycardia (Supplemental Figure 8), while inversely associated with type 2 diabetes.

## Discussion

Here we have derived automated measures of mitral valve annular diameter at systole and diastole for use in discovery of genetic determinants of mitral valve biology from the UK Biobank. We observe a series of genetic loci that appear to be related directly to mitral valve biology or arise from secondary dilation of the mitral valve annulus related to cardiac contractility. A polygenic score derived from these results is predictive of MVP across multiple cohorts, and PheWAS confirmed clinical correlates of mitral valve disease. Importantly, our results do not appear to overlap with previous genetic determinants of mitral valve annular calcification (11) (Supplemental Table 6), suggesting that the estimates of mitral annular diameter and genetic results presented here represent an underlying phenotype that does not originate primarily from degenerative or calcific processes associated with aging or turbulent blood flow (12). Importantly, we observed strong genetic signal in the African/Afro-Caribbean population strata, which differed from the European population, a finding that underscores the desperate need for genetic studies of cardiovascular phenotypes across the diversity of human populations worldwide.

The majority of loci identified appear to be related directly to development and function of the mitral valve apparatus. The *ERBB4* gene is a receptor tyrosine kinase which responds to epidermal growth factors and is recognized to be essential for normal development of cardiac valves in knockout mice (13). Copy number variation encompassing *MCTP2* has been shown to cause congenital heart disease inclusive of mitral atresia or stenosis, and absent endocardial cushions were observed in morpholino knockdowns of *Mctp2* in *Xenopus* embryos (14). The *HDGFL1* locus has been previously implicated in QRS duration and the PR interval (15, 16), which may be epiphenomenon related to the insulating role of the mitral valve annular tissue in electromechanical coupling of the cardiac action potential (17, 18). Among the other loci highlighted, *MCPH1* has been implicated in blood pressure and carotid intima media thickness, while the intergenic 16q21 locus and *MROH7* appear to be without described direct relationships to cardiac, valvular, or vascular phenotypes. The rare variant analyses identified variants proximal to *ST7* and *SLC35F6*, which are genes without recognized cardiovascular phenotypes; however, *NUBPL* is a cause of mitochondrial complex I deficiency syndrome and has been implicated in previous cardiac-related GWAS of heart rate and electrophysiological parameters (19).

Previously identified in GWAS of blood pressure (20, 21) and aortic root diameter (21), the 2 variants in *GOSR2* identified in systole (rs6504673) and diastole (rs533030436), display essentially no linkage in European populations (*r*^2^ = 0.02) and arise from different haplotypes (Supplemental Figure 10). The rs533030436 variant identified in diastolic measures is in strong linkage with rs11874 (*r*^2^ = 0.84), recently identified in congenital cardiovascular malformations (22), as well as rs17608766 (*r*^2^ = 0.76), identified in aortic valve stenosis (23) in the same UK Biobank MRI data set. The cusps and annulus of the mitral valve are composed primarily of endothelial cells and valvular interstitial cells, which may differentiate to myofibroblasts in disease states (24). Our analysis of open chromatin in related cell types (endothelial, cardiomyocyte, fibroblast) is aligned with open chromatin in primary mitral valve tissue. When combined with tissue-specific predictors of 3D genome confirmation, for one example, variants appear to be acting to regulate *GOSR2*. Including the findings presented here, genetic variation in *GOSR2* is now specifically implicated in multiple studies of aortic and mitral valve biology and congenital heart disease, which, when taken together with chromatin accessibility data presented here, strongly suggest a primary role in the formation, growth, and functional maintenance of cardiac valves and vascular tissue.

While the mitral annulus changes in size during the cardiac cycle, it does not have intrinsic contractile properties (25). Some of the loci identified are directly related to cardiac contractility. A missense variant in *BAG3* variant rs2234962 resulting in a cysteine-to-arginine substitution at position 151 (CADD score = 21) is linked with the lead variant rs17099139 (*r*^2^ = 0.74). Both *BAG3* and *TTN* contribute to the function of the contractile apparatus in cardiomyocytes, and rare deleterious variations cause Mendelian forms of dilated cardiomyopathy. Common variation in these genes has been recently recognized to directly influence contractility phenotypes within the UK Biobank cardiac MRI data (8). Additionally, *RBFOX1* has a well-described role in regulating alternative splicing within cardiomyocytes to mediate contractile changes in heart failure and, thus, is likely playing an incidental role in mitral valve biology (26). The influence of these variants upon mitral valve annulus size is likely secondary to their effect upon ventricular contractility; dilation of the mitral valve annulus is a direct consequence of dilation of the LV due to decreased systolic performance.

The polygenic score derived from GWAS of mitral annulus size at systole was associated with mitral valve regurgitation or prolapse across 4 populations, suggesting that the genetic signals derived from anatomical measurements are relevant for the practical purposes of understanding clinical disease. A small amount of mitral valve dysfunction is a clinically insignificant finding or may commonly arise as a consequence of ischemic cardiomyopathy (27, 28). We note that the polygenic risk score for annular diameter was specifically predictive of MVP (Figure 4) and not with mitral regurgitation (Supplemental Figure 7). The use of polygenic scores requires extensive validation before being used clinically for screening and identification of patients at higher risk for adverse remodeling of the left atrium and ventricle, which occurs with progression to significant mitral valve dysfunction. Furthermore, the clinical application of such scores is limited by the notable deficiency of genetic data combined with measurements of cardiac imaging in diverse populations (29).

The clinical associations revealed by PheWAS of mitral annular measures, lead GWAS variants, and polygenic scores are broadly consistent with the genetic correlation results and a current clinical understanding of mitral valve physiology. Decreasing cardiac function may cause dilation of the annulus and mitral valve dysfunction, while mitral valve dysfunction whether primary or secondary due to rheumatic fever or papillary muscle rupture may often result in decreased cardiac function. Interestingly, in both the PheWAS of mitral valve measurements and the polygenic scores, psoriasis displayed a negative association with mitral annular diameter in systole, and mitral valve dysfunction has been reported in patients affected with psoriasis (30). The observational association between MVP and ventricular arrhythmias (31) is supported by the positive association between the PRS for mitral valve annulus diameter in diastole and paroxysmal ventricular tachycardia (Supplemental Figure 8), which may suggest that the mitral valve annulus links these 2 phenotypes. However, we wish to state clearly that while PheWAS results provide context for our findings they do not establish a causal link between mitral valve annular dimensions and the clinical phenotypes identified. Polygenic scores for a specific disease may be strongly correlated with known causal factors for a particular disease (32), and the measurement of mitral valve annular size in systole is genetically correlated with measures of cardiac contractility and volume (Figure 3). As the measurements are derived from a sample of the general population, to some degree they are likely to reflect both environmental stress induced changes to the mitral valve annulus as well as underlying pathological remodeling in early or presymptomatic disease related to cardiac contractility and the mitral valve apparatus itself.

Our study is not without limitations. While we took steps to estimate the error related to image acquisition and our method of automated estimation and to exclude outliers, at each step systematic errors may be introduced which may bias the measurements. Our approach to measuring the mitral annulus was a simple measurement derived from a 2D image; other approaches to quantifying mitral valve morphology may provide a better anatomical substrate for interrogation of genetic and polygenic predictors of disease (33). While the anatomical measure of mitral valve annulus size is predictive of valvular function, GWAS on direct measures of valve function in larger numbers of patients from diverse genetic backgrounds are likely to yield additional important insights into the genetic basis of mitral valve biology and will be necessary for use of polygenic scoring of mitral valve diameter across the diversity of human populations.

In conclusion, the genetic studies of mitral valve annular diameter presented here reveal determinants of mitral valve biology and highlight shared underlying biology with cardiac contractility. Clinical studies highlight both known and previously unrecognized phenotypic associations, and our analyses suggest that polygenic prediction of mitral valve annular diameter may represent an independent risk factor for mitral prolapse. Importantly our findings provide further support evidence for the use of imaging-derived phenotypes as the basis for genetic and clinical studies of cardiovascular health and disease.

## Methods

### UK Biobank cohort and mitral valve measurement from cardiac MRI.

Using a previously described set of algorithms based upon an open-source deep-learning framework (34), we built a U-Net segmentation model with pretrained weights from VGG11, trained on 60 hand-labeled 4-chamber view images, validated on 20 hand-labeled images, and tested on 20 hand-labeled images, which yielded a validation dice score of 91.2% and a the test dice score of 93.8%; these were equivalent to differences observed between expert human annotators (34). Then, the segmentation model was applied to 32,219 4-chamber images from the UK Biobank data set to generate masks for all 4 chambers, including left atrium, LV, right atrium, and right ventricle. After the masks were generated from the trained U-Net segmentation model, another second measuring function was applied to measure the mitral valve annulus diameter, tricuspid valve annulus diameter, interventricular septum length, and atrial septum length. In this measuring function, first the base of the atriums and apex of the ventricles was found along the medial axis of the chambers, and second, the intersecting lines of the atriums and the ventricles were used to locate the annuli of the atrioventricular valves, from which annular diameter could be estimated. Finally, measurements were converted to mm from pixels in units using the metadata in the dicom files.

LV end-systole and LV end-diastole measurements were obtained by selecting the image frame with the largest and smallest estimates of LV volume or left atrial volume as provided by the segmentation algorithm to select the frames best representing LV end-systole and LV end-diastole.

To exclude outliers related to imaging error or methodological inaccuracy, automated measurements were plotted relative to body surface area with standard measures of quality control. We excluded measures ± 3 standard deviations for each of the 4 measures. A trained clinician performed manual annotation of 100 randomly selected images spanning the cardiac cycle to gauge the accuracy of the estimate of mitral valve annulus diameter. Manual annotation was performed blinded to automated measures or anthropometric characteristics. The percentage difference between automated and manual measurements ([automated measure – manual measure]/manual measure) was examined for systematic relationships to anthropomorphic predictors (age in years, body surface area, genetic sex), imaging acquisition error (posterior or anterior deviation of the imaging plane from the ideal), and cardiac cycle (ventricular systole or ventricular diastole) using standard linear modeling.

### GWAS and annotation.

The UK Biobank data release available at the time of analysis included genotypes for 488,377 participants, obtained through either the custom UK Biobank Axiom array or the Affymetrix Axiom Array. Genotypes were imputed to the TOPMed panel (version 5) of the Michigan imputation server. Only variants with MAF greater than or equal to 0.01 and minor allele count greater than or equal to 5, and variants that have Hardy-Weinberg equilibrium exact test *P* value over 1× 10^–20^ in the entire MRI data set and an empirical-theoretical variance ratio (Mach *r*^2^) threshold above 0.3, were included. The main GWAS was conducted on the largest subset of participants with MRI data from the largest unrelated European ancestry cohort defined using the variable in.white.British.ancestry.subset in the file ukb_sqc_v2.txt provided as part of the UKB data release (*n* = 32,220 individuals with estimates derived from imaging data). To replicate the findings, we separated the global data set into a discovery set and a replication set, which included at least 22,124 and 4,950 participants, separately. To further replicate the findings from the first stage, we included 3 independent sets of individuals of other ethnic backgrounds with cardiac MRI who were not included in the discovery set, including African/Afro-Caribbean (*n* = 222, age = 49.6 ± 7.0 years), East Asian (*n* = 85, age = 49.2 ± 5.5 years), and South Asian (*n* = 368, age = 52.1 ± 7.9 years) strata. Examination of those samples according the genetic principal components showed that many were mostly of European ancestry and were unrelated (Supplemental Figure 10). Association tests were performed using linear regression PLINK2 (v2.00a2LM) additive model (35, 36), including sex, age, adjusted body surface area, and principal components 1–10 as covariates. Locuszoom was used to generate regional association plots. For a simple association test of rare variants with a MAF of less than 1% and imputation quality (Mach *r*^2^) greater than 0.8, we combined discovery and replication sets into a single analysis of 4,485,863 additional variants for both the measurements of mitral valve annular diameter in systole and diastole.

Variants were annotated using FUMA, which required liftover conversion of variant coordinates from GRCh38/hg38 to GRCh37/hg19 as input, based on the 1000G Phase3 EUR reference panel population. The independent significant SNPs were identified by the LD *r*2 of 0.6. All SNPs in LD with any of independent significant SNPs with LD of *r*2 greater or equal to 0.6 were annotated to eQTL (GTEx v8; heart atrial appendage and heart LV), CADD, RDB, and the GWAS catalog. LD between pairs of variants was examined and reported in *r*^2^ using the web-based LDPair tool (release April 29, 2020; https://ldlink.nci.nih.gov). We used eMAGMA v1.08 to associate genetic determinants of mitral valve annulus size at systole and diastole with specific genes at the tissue level for 4 relevant tissues (aorta, LV, atrial appendage, and coronary artery). To model LD structure, we used phase 3 of 1000 Genomes reference for the European population, and multiple-hypothesis testing was accounted for using 10,000 adaptive permutations (--adap-permp = 10,000).

We analyzed open chromatin regions from mitral valves of 5 patients undergoing mitral valve replacement (Supplemental Table 7) as previously described (37). Variants in high LD (*r*^2^ ≥ 0.5 in European populations) with lead SNPs were retrieved using LDproxy function of LDlinkR package (38). Variants overlapping open chromatin in at least 2 mitral valve samples were reported. We tested overlap of variants with assay for transposase-accessible chromatin with sequencing (ATAC-Seq) peaks (narrowpeak from MACS2 output + 100 bp on each side) using bedtools (v2.29.0) annotate function. Read density profiles from 2 valve data sets, 2 fibroblast cell lines, and 2 heart data sets from ENCODE were calculated as previously described (39) and visualized using Integrated Genome Browser (v9.1.4). We also visualized H3K27ac histone mark read density profiles of ENCODE data sets generated from heart LV (ENCSR702OVJ), ascending aorta (ENCSR982QIF), or primary fibroblasts (ENCSR000APN).

For an analysis of predicted enhancer mapping, we considered the set of variants in LD (*r*^2^ ≥ 0.8) with the lead variants in 1000 Genomes and overlapped these variants with predicted enhancers in 131 cell types identified by the ABC model, which combines measurements of enhancer activity (based on ATAC-Seq, DNase-Seq, and H3K27ac ChIP-Seq) with measurements of 3D contact frequencies (based on Hi-C) (40, 41). We examined DNase-Seq and ATAC-Seq data across a range of cardiovascular cell types from the ENCODE Project (42). Finally, we examined sequence motif predictions for identified variants using a database of transcription factor binding site motifs (43).

### LDSC and genetic correlation analyses.

To calculate genetic correlation between polygenic risk score of mitral valve annular size and other related phenotypes, we obtained summary statistics for cardiac MRI–derived LV measurements (LV end-diastolic volume [LVEDV], LV end-systolic volume [LVESV], stroke volume [SV], the body surface area indexed versions for cardiovascular traits [LVEDVi, LVESVi, and SVi], and LV ejection fraction, ref. 8) as well as atrial fibrillation (44), nonischemic cardiomyopathy (45), heart failure (46), heart failure using UK Biobank data (45), hypertension (47), PR interval (16), myocardial infarction and coronary artery disease (48), heart rate (49), and MVP (6). Using these data, we performed LD score regression (50) based on the reformatted summary statistics filtered to HapMap3.

### PheWAS.

We performed PheWAS to highlight clinical associations with up to 1240 ICD-10 aggregated clinical phenotypes defined as phecodes (51). Methods were as previously described (23). For PheWAS results reported here, we excluded phenotypes with less than 50 individuals (for continuous traits) or less than 50 cases (for binary-coded traits), and we corrected for multiple testing using a Bonferroni adjustment. We performed PheWAS for the mitral annulus measurements among individuals with the MRI data (*n* = 32,219) and PheWAS for the GWAS significant variants and for the polygenic scores (described below) among individuals without the MRI data (*n* = 308,683 participants).

### Polygenic prediction of MVP.

Summary statistics from the mitral valve annular diameter GWAS were used to compute a polygenic score for the remaining set of unrelated individuals of European ancestry. We separated the European ancestry replication set further, dividing it into a training (*n* = 4000) and validation subset (*n* = 1000). We trained the best PRS using the summary statistics from the discovery set in the training subset using PRSice-2 (52) and validated the maximally performing PRS (measured by *r*^2^) using the validation set. Only variants with MAF greater than 0.01 were used to calculate the PRS, as well as the variants in LD (*r*^2^ ≥ 0.8). We also validated the best PRS in the MVP cohorts from France, Nantes, UK Biobank, and Penn Medicine BioBank, which include 1007 cases and 1469 controls, 479 cases and 862 controls, 434 cases and 4527 controls, and 144 cases and 20890 controls, respectively. The definition of MVP in the Penn Medicine BioBank was determined using ICD-10 code I34.1 (nonrheumatic MVP), while excluding individuals with codes I05.1, I05.2, and 394.1, which incorporate or do not specifically exclude mitral valve dysfunction related to rheumatic heart disease. The associations between MVP and the best PRS of mitral valve annular diameter at the MVP individual level were conducted using logistic regression. The sum of best PRS for MVP and MR at the individual level was estimated by PLINK2.

### Statistics.

The standard genome-side significance threshold of *P* ≤ 5 × 10–8 for GWAS were used for genomic studies. Other significance thresholds for LDSC and PRS analyses were categorized as significant using a Bonferroni-corrected *P* < 0.05 per a specific number of tests. Statistical details for each individual analysis can be found in the relevant methods subsection.

### Study approval.

This work employed deidentified human data from the UK Biobank. Ethical approval was granted by the NHS National Research Ethics Service (ref: 11/NW/0382) (Stockport, United Kingdom).

## Author contributions

MY provided study design, data analysis for GWAS and PRS, and manuscript writing. CT provided study design, data analysis for PheWAS, methodological contributions for PRS, and manuscript writing. AG contributed data analysis, methodological primary mitral valve tissue analyses, and manuscript writing. KX provided data analysis of machine-learning models. HT provided data analysis of machine-learning models. CD provided analysis of French mitral valve data sets. TLT contributed relevant data. MF supervised machine-learning models. RJ provided analysis of mitral valve PRS data. NLT provided analysis of mitral valve PRS data. DA provided analysis and fine mapping of genomic context. CJM provided analysis and fine mapping of genomic context. JME contributed methodological supervision of fine mapping work. SMD supervised mitral valve PRS data analyses. NBN supervised mitral valve PRS data and primary mitral valve tissue analyses. JRP provided study design and conception as well as manuscript writing.

## Supplementary Material

Supplemental data

## Figures and Tables

**Table 1:**
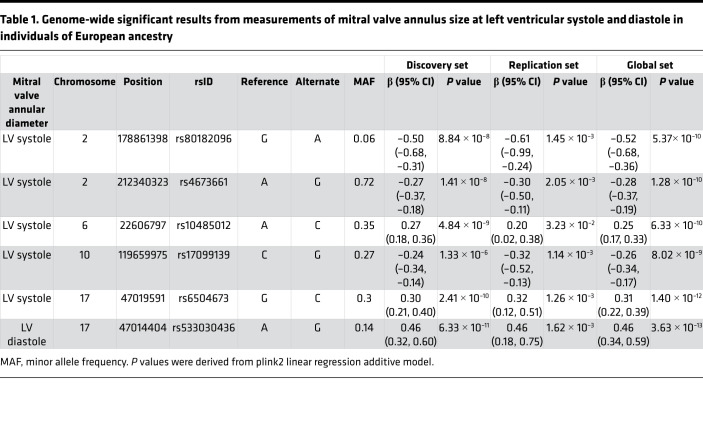
Genome-wide significant results from measurements of mitral valve annulus size at left ventricular systole and diastole in individuals of European ancestry

## Version 1 02/08/2022

Electronic publication

## References

[1] Garbi M, Monaghan MJ (2015). Quantitative mitral valve anatomy and pathology. Echo Res Pract.

[2] de Vlaming A (2012). Atrioventricular valve development: new perspectives on an old theme. Differentiation.

[3] Combs MD, Yutzey KE (2009). Heart valve development: regulatory networks in development and disease. Circ Res.

[4] Nishimura RA (2017). 2017 AHA/ACC Focused update of the 2014 AHA/ACC guideline for the management of patients with valvular heart disease: a report of the American College of Cardiology/American Heart Association Task Force on clinical practice guidelines. Circulation.

[5] Hagège AA (2015). Dynamic changes of the mitral valve annulus: new look at mitral valve diseases.. Circ Cardiovasc Imaging.

[6] Toomer KA (2019). Primary cilia defects causing mitral valve prolapse. Sci Transl Med.

[7] Yu M (2019). Genome-wide association study-driven gene-set analyses, genetic, and functional follow-up suggest GLIS1 as a susceptibility gene for mitral valve prolapse. Circ Genom Precis Med.

[8] Pirruccello JP (2020). Analysis of cardiac magnetic resonance imaging in 36,000 individuals yields genetic insights into dilated cardiomyopathy. Nat Commun.

[10] Mihaila S (2015). Normal mitral annulus dynamics and its relationships with left ventricular and left atrial function. Int J Cardiovasc Imaging.

[11] Thanassoulis G (2013). Genetic associations with valvular calcification and aortic stenosis. N Engl J Med.

[12] Levine RA (2015). Mitral valve disease--morphology and mechanisms. Nat Rev Cardiol.

[13] Iwamoto R (2003). Heparin-binding EGF-like growth factor and ErbB signaling is essential for heart function. Proc Natl Acad Sci U S A.

[14] Lalani SR (2013). MCTP2 is a dosage-sensitive gene required for cardiac outflow tract development. Hum Mol Genet.

[15] Norland K (2019). Sequence variants with large effects on cardiac electrophysiology and disease. Nat Commun.

[16] Ntalla I (2020). Multi-ancestry GWAS of the electrocardiographic PR interval identifies 202 loci underlying cardiac conduction. Nat Commun.

[17] Saremi F (2017). Fibrous skeleton of the heart: anatomic overview and evaluation of pathologic conditions with CT and MR imaging. Radiographics.

[18] Knecht S (2008). Relationship between perimitral and peritricuspid conduction times. Heart Rhythm.

[19] Åberg K (2012). Genome-wide association study of antipsychotic-induced QTc interval prolongation. Pharmacogenomics J.

[20] Simino J (2014). Gene-age interactions in blood pressure regulation: a large-scale investigation with the CHARGE, global BPgen, and ICBP consortia. Am J Hum Genet.

[21] Ehret GB (2011). Genetic variants in novel pathways influence blood pressure and cardiovascular disease risk. Nature.

[22] Lahm H (2021). Congenital heart disease risk loci identified by genome-wide association study in European patients. J Clin Invest.

[23] Córdova-Palomera A (2020). Cardiac imaging of aortic valve area from 34 287 UK biobank participants reveals novel genetic associations and shared genetic comorbidity with multiple disease phenotypes. Circ Genom Precis Med.

[24] Arounlangsy P (2004). Histopathogenesis of early-stage mitral annular calcification. J Med Dent Sci.

[25] Silbiger JJ, Bazaz R (2020). The anatomic substrate of mitral annular contraction. Int J Cardiol.

[26] Gao C (2016). RBFox1-mediated RNA splicing regulates cardiac hypertrophy and heart failure. J Clin Invest.

[27] Cebeci BS (2004). Echocardiographical characteristics of healthy young subjects with physiological mitral regurgitation. J Int Med Res.

[28] Filsoufi F (2006). Physiologic basis for the surgical treatment of ischemic mitral regurgitation. Am Heart Hosp J.

[29] Martin AR (2019). Clinical use of current polygenic risk scores may exacerbate health disparities. Nat Genet.

[30] Gorga E (2018). Echocardiographic evaluation of diastolic dysfunction in young and healthy patients with psoriasis: a case-control study. Monaldi Arch Chest Dis.

[31] Basso C (2015). Arrhythmic mitral valve prolapse and sudden cardiac death. Circulation.

[32] Ibanez L (2019). Overlap in the genetic architecture of stroke risk, early neurological changes, and cardiovascular risk factors. Stroke.

[33] Garg P (2020). Assessment of mitral valve regurgitation by cardiovascular magnetic resonance imaging. Nat Rev Cardiol.

[34] Bai W (2018). Automated cardiovascular magnetic resonance image analysis with fully convolutional networks. J Cardiovasc Magn Reson.

[35] Purcell S (2007). PLINK: a tool set for whole-genome association and population-based linkage analyses. Am J Hum Genet.

[36] https://www.cog-genomics.org/plink/2.0/.

[37] Kyryachenko S (2021). Chromatin accessibility of human mitral valves and functional assessment of MVP risk loci. Circ Res.

[38] Myers TA (2020). LDlinkR: an R package for rapidly calculating linkage disequilibrium statistics in diverse populations. Front Genet.

[40] Fulco CP (2019). Activity-by-contact model of enhancer-promoter regulation from thousands of CRISPR perturbations. Nat Genet.

[41] Nasser J (2021). Genome-wide enhancer maps link risk variants to disease genes. Nature.

[42] Dunham I (2012). An integrated encyclopedia of DNA elements in the human genome. Nature.

[43] Kulakovskiy IV (2018). HOCOMOCO: towards a complete collection of transcription factor binding models for human and mouse via large-scale ChIP-Seq analysis. Nucleic Acids Res.

[44] Roselli C (2018). Multi-ethnic genome-wide association study for atrial fibrillation. Nat Genet.

[45] Aragam KG Phenotypic refinement of heart failure in a national biobank facilitates genetic discovery. Circulation.

[46] Shah S (2020). Genome-wide association and Mendelian randomisation analysis provide insights into the pathogenesis of heart failure. Nat Commun.

[47] Wojcik GL (2019). Genetic analyses of diverse populations improves discovery for complex traits. Nature.

[48] Nikpay M (2015). A comprehensive 1,000 genomes-based genome-wide association meta-analysis of coronary artery disease. Nat Genet.

[49] den Hoed M (2013). Identification of heart rate–associated loci and their effects on cardiac conduction and rhythm disorders. Nat Genet.

[50] Bulik-Sullivan B (2015). LD Score regression distinguishes confounding from polygenicity in genome-wide association studies. Nat Genet.

[51] Wu P (2019). Mapping ICD-10 and ICD-10-CM Codes to phecodes: workflow development and initial evaluation. JMIR Med Inform.

[52] Euesden J (2015). PRSice: polygenic risk score software. Bioinformatics.

