# Supplementary material for: Computational estimates of annular diameter reveal genetic determinants of mitral valve function and disease
Source: JCI Insight. 2022 Feb 8;7(3):e146580. doi: 10.1172/jci.insight.146580 (PMC8855800; doi:10.1172/jci.insight.146580)
Supplement: Supplemental data [file jciinsight-7-146580-s273.pdf]

Supplementary Figures and Tables for Yu et al.

(In order of appearance in the main manuscript text)

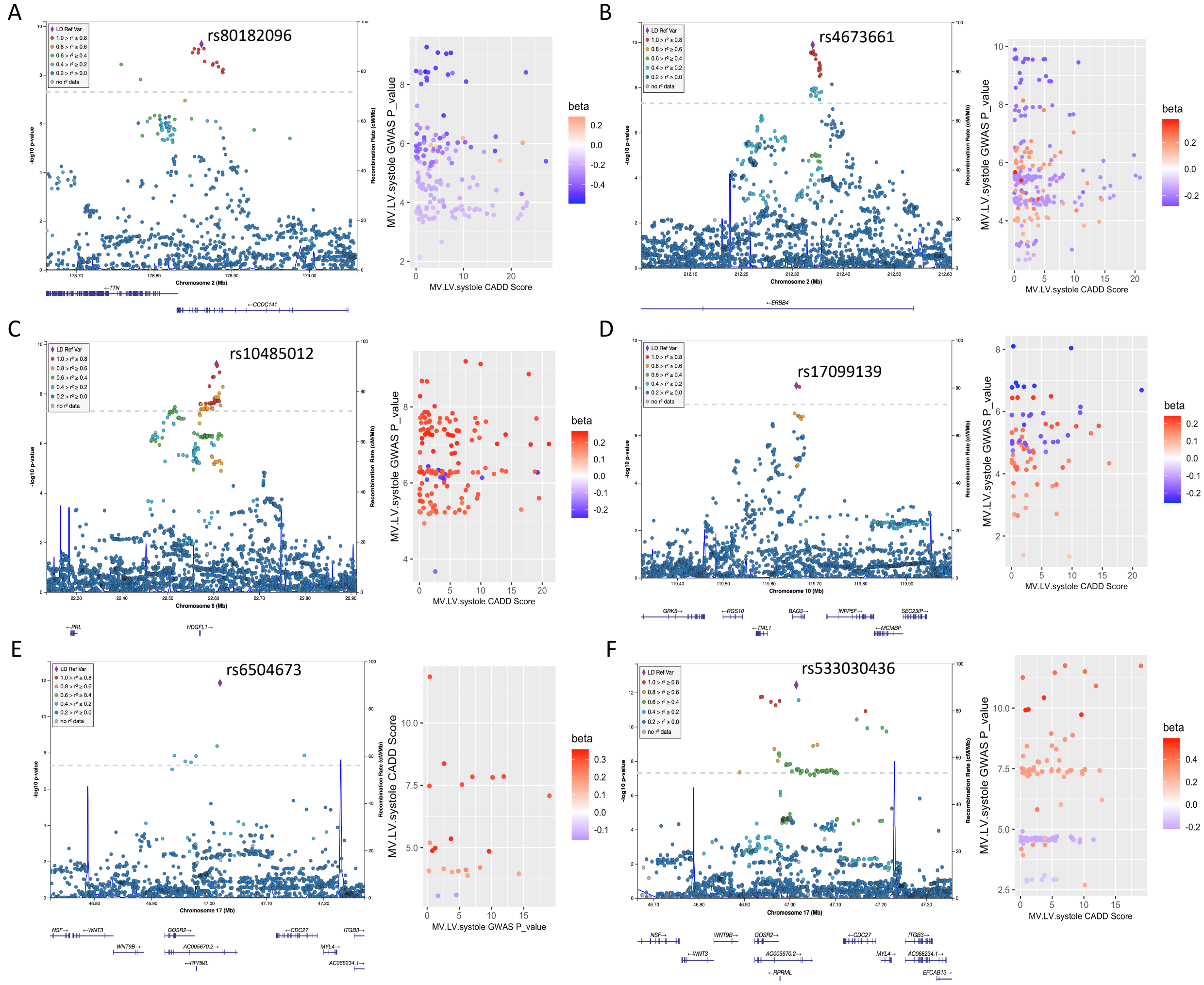

Figure S1. Locuszoom plots for mitral annular diameter in systole and diastole amongst European ancestry population strata. CADD scores are plotted on the righthand panels. Panels A and B represent genetic signal originating from genes related to contractility (*TTN* and *BAG3*). Panels C and D represent genetic signal arising from *ERBB4* and *HDGFL1* which are likely to capture aspects of underlying mitral valve biology. Panels E and F show two separate haplotypes for *GOSR2* which were detected for systole and diastole separately. Panels A-E show the signals from MV at LV.systole and panel F show MV at LV.diastole.

Table S1. Rare-variant associations with mitral-valve annular diameter.

Phenotype	Chr	Position (hg38)	rsid	Ref allele	Alt allele	MAF	MACH R2	Beta	Standard Error	p-value	Genomic context
MV annulus **diastole**	chr14	31521207	rs182990429	C	T	0.00108	0.80082	-4.4545	0.814659	4.59E-08	Intron of *NUBPL*
MV annulus **systole**	chr2	26757360	rs550914510	A	G	0.003775	0.897673	-2.141	0.365979	4.92E-09	Intergenic, 6925 bp upstream of *SLC35F6*
MV annulus **systole**	chr7	117024524	rs571176355	A	C	0.002199	0.892558	-2.6528	0.480472	3.39E-08	Intron of *ST7*

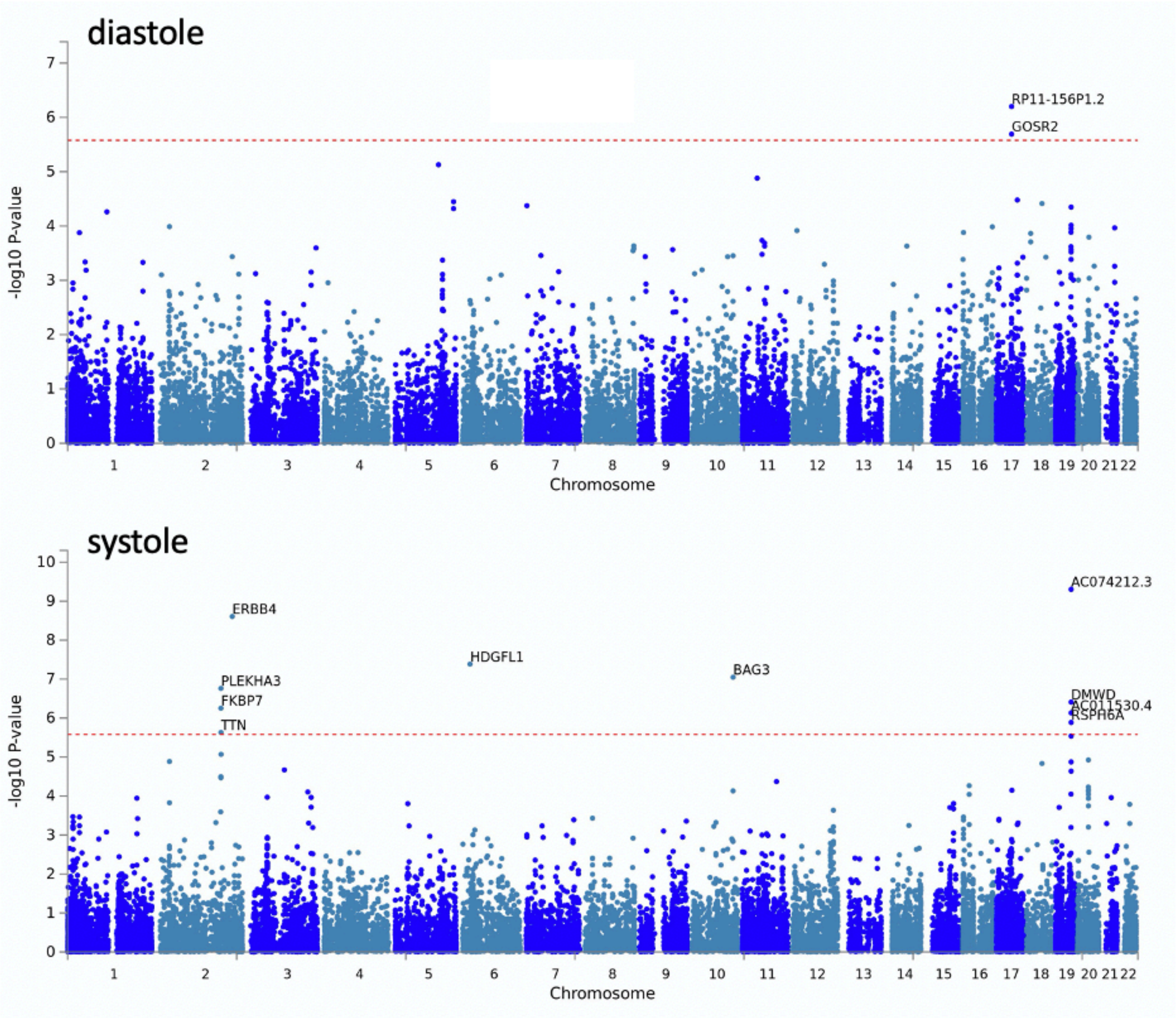

Figure S2. Gene based plots of eMAGMA localization analyses. The primary signal for mitral valve annular diameter in diastole on chromosome 17 localizes to *GOSR2* and the lead variant rs17608766 is not an eQTL in left ventricle, atrial appendage, aorta, or coronary artery tissue suggesting the need for mitral valve-specific analyses. The eMAGMA analysis localizes signals for mitral valve annular diameter in systole to genes related to myocardial function (*TTN, BAG3)* as well as novel genes (*ERBB4, DMWD, HDGFL1*) also without strong eQTL support in tissues sampled from nearby to the mitral valve (left ventricle, atrial appendage, aorta, or coronary artery) suggesting the necessity of tissue specific ATACseq and molecular analyses (Figure 2).

Lead Variants				Variants overlapping MV open chromatin regions					Top eQTLs (fibroblasts, GTEx v8)	
rsID	Chr	Pos (hg38)	P-value	rsID	Pos (hg38)	LD (r^2)	Correlated alleles	p-value	Gene	p-value
rs80182096	chr2	178 861 398	3.8×10^{-9}	rs60105920	178 883 241	0.86	G=G,A=A	1.2×10^{-8}	*FKBP7*	1.6×10^{-5}
rs4673661	chr2	212 340 323	1.0×10^{-8}							
rs10485012	chr6	22 606 797	5.0×10^{-9}							
rs17099139	chr10	119 659 975	4.1×10^{-8}							
rs6504673	chr17	47 019 591	2.0×10^{-9}	rs71365052	47 205 379	0.59	G=TG,C=-	6.5×10^{-4}	*GOSR2*	2.9×10^{-8}
rs533030436	chr17	47 014 404	4.9×10^{-11}	rs17608766	46 935 905	0.76	A=T,G=C	2.3×10^{-10}	*GOSR2*	4.3×10^{-8}

Table S2. Results from trans-ethnic meta-analysis and individual population-strata analysis of mitral valve annulus diameter measured at systole and diastole.

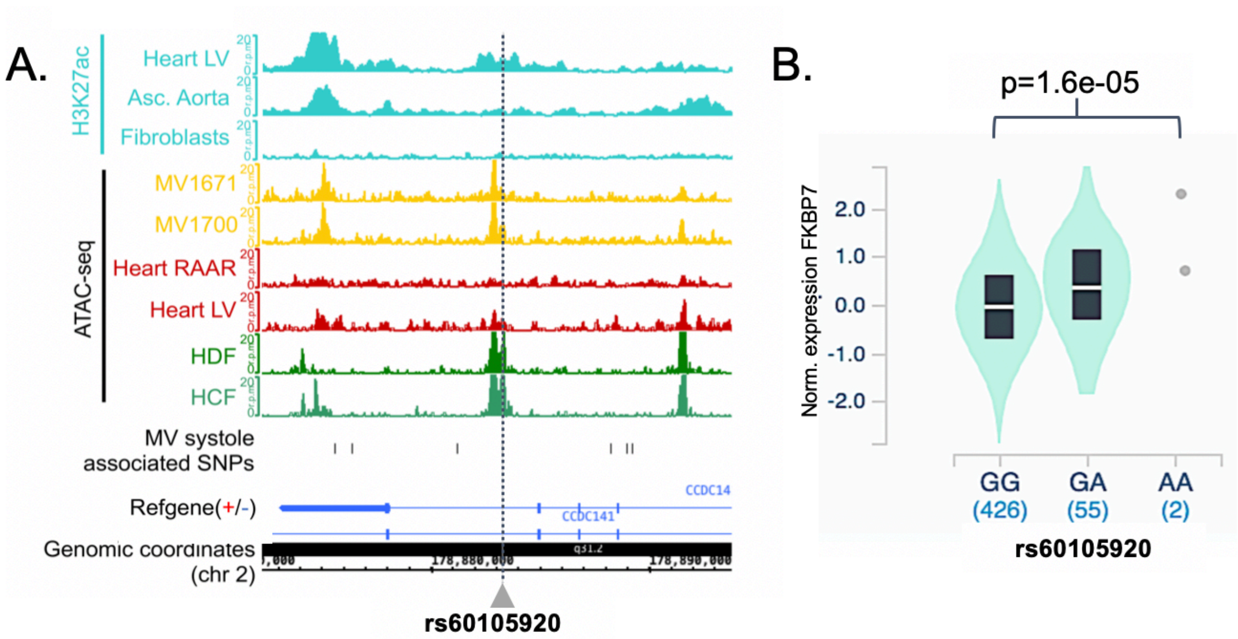

Figure S3. Variation associated with mitral valve annular diameter in systole is related to FKBP7 A. Genome browser visualization of ATAC-Seq/Histone ChIP read densities (in reads/million, r.p.m.) at chromosome 2 CCDC141 locus in the region surrounding rs60105920. **B.** Violin plot representation of genotype-expression association between rs60105920 and FKBP7 in cultured fibroblasts samples from GTEx (v8 release). Abbreviations MV: Normal Valves. HDF: human dermal fibroblasts. HCF: human cardiac fibroblasts. Heart LV: Heart left ventricle. Heart RAAR: Heart right atrium auricular region. Asc.Aorta: Ascending Aorta.

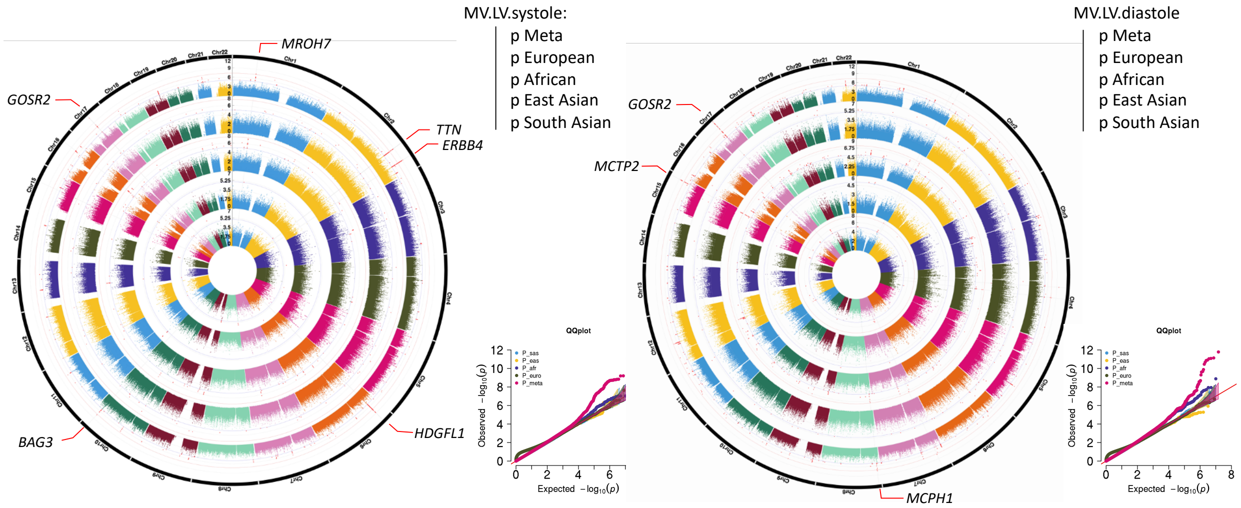

Figure S4. Circos plots for of the trans-ancestry meta-analysis of mitral annular diameter in systole and diastole. Systolic measurements are on the left-hand plot, diastolic measurements are on the righthand plots, with quantile-quantile plots inset. From outer to inner are plotted South Asian, East Asian, African/Afro-Caribbean, European, and the meta-analysis.

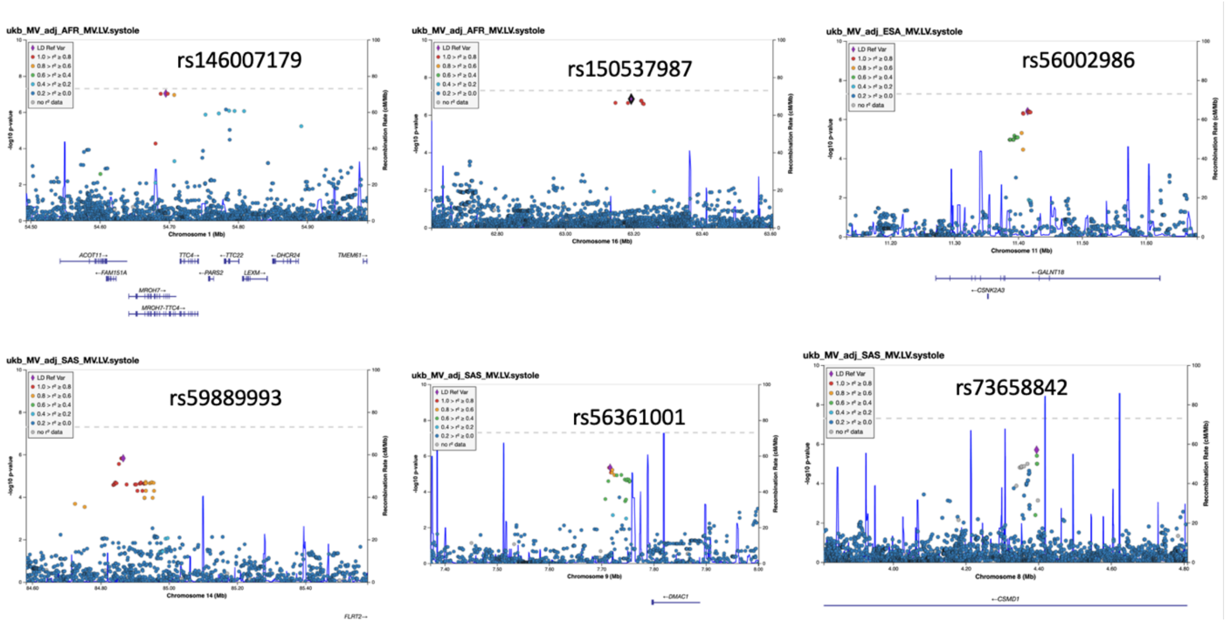

Figure S5. Locuszoom plots for mitral annular diameter in systole amongst African/Afro-Caribbean, East Asian, and South Asian population strata.

				Meta_analysis								European GWAS				AFR GWAS				EAS GWAS				SAS GWAS			
Measure	Population lead	CHR	POS38	Allele1	Allele2	Freq1	Effect	StdErr	P.value	Direction	HetPVal	A1_euro	1_FREQ_eu	BETA_euro	P_euro	A1_afr	A1_FREQ_afr	BETA_afr	P_afr	A1_eas	1_FREQ_ea	BETA_eas	P_eas	A1_sas	1_FREQ_sa	BETA_sas	P_sas
Diastole	EURO	17	47014404	a	g	0.86	-0.46	0.07	1.65E-12	--?+	0.48	G	0.14	0.46 [0.34, 0.59]	3.63E-13	G	0.02	1.77 [-2.27, 5.82]	0.39	NA	NA	NA	NA	G	0.04	-0.42 [-2.09, 1.25]	0.62
	AFR	15	94387693	t	g	0.94	6.16	1.04	2.74E-09	?+??	1	NA	NA	NA	NA	G	0.06	-6.16 [-8.20, -4.13]	1.15E-08	NA	NA	NA	NA	NA	NA	NA	NA
	AFR	8	5721520	c	g	0.98	9.86	1.76	2.24E-08	?+??	1	NA	NA	NA	NA	G	0.02	-9.86 [-13.32, -6.41]	7.04E-08	NA	NA	NA	NA	NA	NA	NA	NA
	AFR	4	18770142	a	g	0.07	0.00	0.09	0.9718	-++?	1.61E-06	A	0.07	-0.02 [-0.19, 0.14]	0.79	A	0.01	10.35 [6.42, 14.28]	5.81E-07	A	0.08	0.08 [-2.19, 2.36]	0.94	NA	NA	NA	NA
	EAS	1	94020850	t	c	0.75	-0.07	0.05	0.1607	----	2.10E-05	C	0.25	0.05 [-0.05, 0.15]	0.30	C	0.31	0.07 [-0.94, 1.09]	0.89	C	0.11	4.88 [2.94, 6.81]	5.28E-06	C	0.23	0.40 [-0.35, 1.14]	0.294992
	EAS	1	90726025	a	g	0.77	0.05	0.05	0.3833	++-+	4.48E-06	G	0.23	-0.05 [-0.16, 0.05]	0.29	G	0.06	-1.69 [-3.75, 0.37]	0.11	G	0.20	3.25 [1.94, 4.57]	7.10E-06	G	0.28	-0.41 [-1.14, 0.31]	0.261362
	SAS	1	225481864	t	c	0.05	-0.20	0.10	0.05478	-??-	5.39E-08	T	0.05	-0.14 [-0.34, 0.06]	0.16	NA	NA	NA	NA	NA	NA	NA	NA	T	0.02	-5.80 [-7.82, -3.77]	4.09E-08
	SAS	7	43946004	a	g	0.34	-0.05	0.05	0.2725	-++-	1.88E-05	A	0.33	-0.02 [-0.12, 0.07]	0.61	A	0.27	0.51 [-0.64, 1.65]	0.39	A	0.57	0.99 [-0.63, 2.60]	0.23	A	0.55	-1.47 [-2.06, -0.87]	1.94E-06
	SAS	11	106362327	a	c	0.99	7.77	1.61	1.40E-06	???+	1	NA	NA	NA	NA	NA	NA	NA	NA	NA	NA	NA	NA	C	0.01	-7.77 [-10.92, -4.62]	2.04E-06
	SAS	18	66746251	a	g	0.94	-0.04	0.09	0.6595	-??+	5.81E-07	G	0.06	0.07 [-0.11, 0.24]	0.447391	NA	NA	NA	NA	NA	NA	NA	NA	G	0.01	-7.52 [-10.49, -4.55]	1.06E-06
Systole	EURO	17	47019591	c	g	0.30	0.31	0.04	7.66E-12	+???	1	C	0.30	0.31 [0.22, 0.39]	1.40E-12	NA	NA	NA	NA	NA	NA	NA	NA	NA	NA	NA	NA
	EURO	2	212340323	a	g	0.28	0.28	0.04	6.42E-10	+?-?	0.39	G	0.72	-0.28 [-0.36, -0.19]	1.28E-10	NA	NA	NA [NA, NA]	NA	G	0.81	0.60 [-1.38, 2.57]	0.56	NA	NA	NA	NA
	EURO	2	178861398	a	g	0.06	-0.51	0.09	1.98E-09	--??	0.69	A	0.06	-0.52 [-0.68, -0.36]	5.37E-10	A	0.15	-0.27 [-1.50, 0.97]	0.67	NA	NA	NA	NA	NA	NA	NA	NA
	EURO	6	22606797	a	c	0.65	-0.26	0.04	1.45E-09	-+?-	0.74	C	0.35	0.25 [0.17, 0.33]	6.33E-10	C	0.16	-0.06 [-1.29, 1.17]	0.92	NA	NA	NA	NA	C	0.18	0.49 [-0.29, 1.28]	0.22
	EURO	10	119659975	c	g	0.73	0.26	0.05	2.51E-08	+???	1	G	0.27	-0.26 [-0.34, -0.17]	8.02E-09	NA	NA	NA	NA	NA	NA	NA	NA	NA	NA	NA	NA
	AFR	1	54695412	t	c	0.02	-10.12	1.83	3.05E-08	?-??	1.00E+00	NA	NA	NA	NA	T	0.02	-10.12 [-13.70, -6.54]	9.29E-08	NA	NA	NA	NA	NA	NA	NA	NA
	AFR	16	63193408	t	c	0.98	8.31	1.52	4.96E-08	?+??	1	NA	NA	NA	NA	C	0.02	-8.31 [-11.29, -5.32]	1.42E-07	NA	NA	NA	NA	NA	NA	NA	NA
	AFR	7	110364350	t	c	0.02	-3.12	1.02	0.002157	?-?-	3.03E-05	NA	NA	NA	NA	T	0.01	-9.87 [-13.62, -6.12]	5.72E-07	NA	NA	NA	NA	T	0.02	-0.46 [-2.81, 1.89]	0.70
	EAS	11	11414686	t	c	0.08	0.11	0.08	0.1524	++++	1.01E-06	T	0.08	0.05 [-0.09, 0.20]	0.48	T	0.02	1.50 [-2.27, 5.26]	0.44	T	0.22	3.56 [2.31, 4.80]	3.84E-07	T	0.09	0.25 [-0.82, 1.32]	0.65
	SAS	14	84853789	a	g	0.98	0.17	0.16	0.3036	-+?+	5.35E-06	G	0.01	0.04 [-0.29, 0.36]	0.82	G	0.16	-1.00 [-2.23, 0.22]	0.11	NA	NA	NA	NA	G	0.02	-4.56 [-6.44, -2.69]	2.77E-06
	SAS	9	7714512	a	c	0.02	-0.30	0.13	0.02447	-+?-	2.80E-05	A	0.02	-0.25 [-0.51, 0.01]	0.06	A	0.08	0.84 [-0.83, 2.50]	0.33	NA	NA	NA	NA	A	0.02	-4.73 [-6.72, -2.74]	4.52E-06
	SAS	8	4394753	a	c	0.98	-6.23	1.29	1.32E-06	???-	1	NA	NA	NA	NA	NA	NA	NA	NA	NA	NA	NA	NA	C	0.02	6.23 [3.71, 8.75]	1.99E-06

Table S3. Results from trans-ethnic meta-analysis and individual population-strata analysis of mitral valve annulus diameter measured at systole and diastole.

CHR	POS	ID	REF	A1	A1_FREQ	MV.LV.diastole		MV.LV.systole	
						Beta [95%CI]	P-value	Beta [95%CI]	P-value
2	178861398	rs80182096	G	A	0.06	-0.40 [-0.58, -0.21]	2.24×10^{-05}	-0.52 [-0.68, -0.36]	5.37×10^{-10}
2	212340323	rs4673661	A	G	0.72	-0.20 [-0.30, -0.11]	2.85×10^{-05}	-0.28 [-0.37, -0.19]	1.28×10^{-10}
6	22606797	rs10485012	A	C	0.35	0.18 [0.08, 0.27]	1.45×10^{-04}	0.25 [0.17, 0.33]	6.33×10^{-10}
10	119659975	rs17099139	C	G	0.27	-0.17 [-0.26, -0.07]	7.53×10^{-04}	-0.26 [-0.34, -0.17]	8.02×10^{-09}
17	47014404	rs533030436	A	G	0.14	0.46 [0.34, 0.59]	3.63×10^{-13}	0.34 [0.22, 0.45]	4.26×10^{-09}
17	47019591	rs6504673	G	C	0.30	0.34 [0.24, 0.43]	2.76×10^{-12}	0.31 [0.22, 0.39]	1.4×10^{-12}

Table S4. Comparison of signal from lead variants from individual measurements of the mitral annulus in both ventricular systole and diastole.

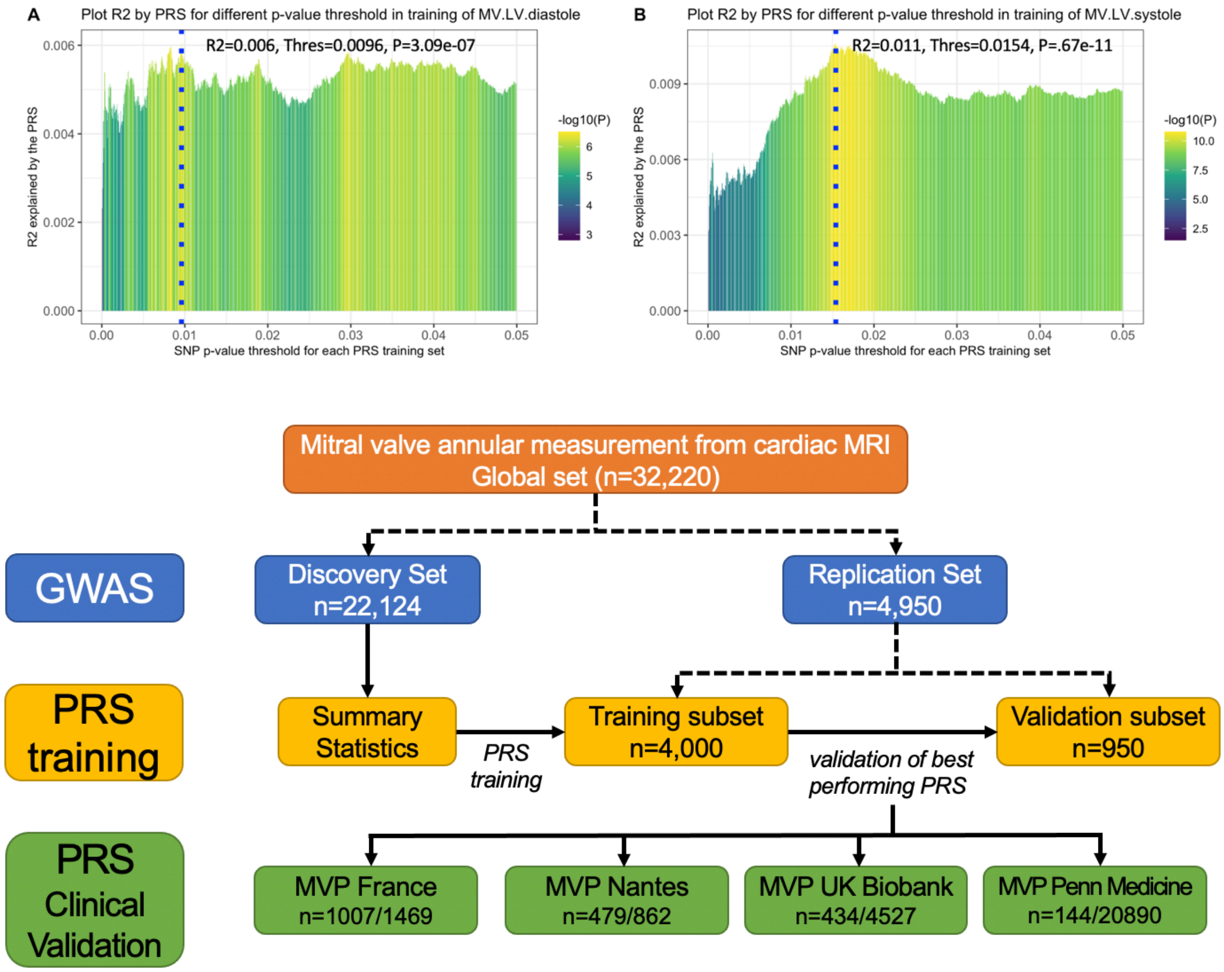

Figure S6. Schema for creation and validation of polygenic risk scores for mitral valve annular diameter. A. Thresholding of p-values and R2 captured for Mitral valve annular diameter at systole and diastole. Note that R2 is relatively uniform across a variety of p-value thresholds for the diastolic measurement **B.** the systolic measurement reaches a clear maximal value for R2 at a p-value inclusion threshold of 0.0154. **C.** Data flow dividing the global set into discovery and replication sets permits the subdivision of the replication set into a PRS-training and PRS-validation subsets for both mitral valve annular diameter in systole and diastole. In this schema no individual is used twice during the creation of the polygenic scores, which are then further validated externally in the four external datasets of mitral valve prolapse (MVP).

	Training	Validation in 1000 mv biobank	Validation on MVP-France	Validation on MVP-Nantes	Validation on MVP-UKB
MV.LV.diastole	R2=0.0061, Thres=0.0096, P=3.09e-07, SNPs=19,589	R2=0.0055, Thres=0.0096, P=0.0131, SNPs=19,559	R2=0.0023, P=0.0327, SNPs=18,760	R2=0.0024, P=0.1158, SNPs=18,521	R2=0.0043, P=0.0019, SNPs=19,444
MV.LV.systole	R2=0.0106, Thres=0.0154, P=1.67e-11, SNPs= 31,912	R2=0.0031, Thres=0.0154, P=0.0665, SNPs=31,873	R2=0.0074, P=0.00011, SNPs=30,497	R2=0.0052, P=0.0213, SNPs=30,077	R2=0.0069, P=0.000087, SNPs=31,663

Table S5. Selected p-value thresholds and R2 and number of SNPs yielded for polygenic scoring of mitral valve annular diameter at systole and diastole within different cohorts.

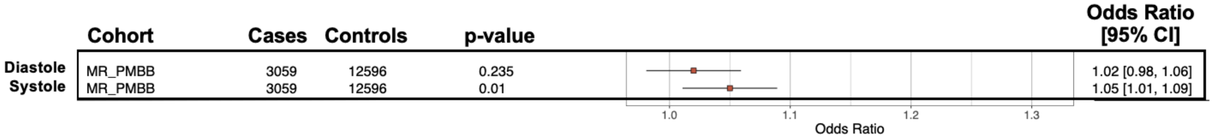

Figure S7. Forest plots predictive of polygenic prediction of mitral valve regurgitation in the Penn Medicine Biobank.

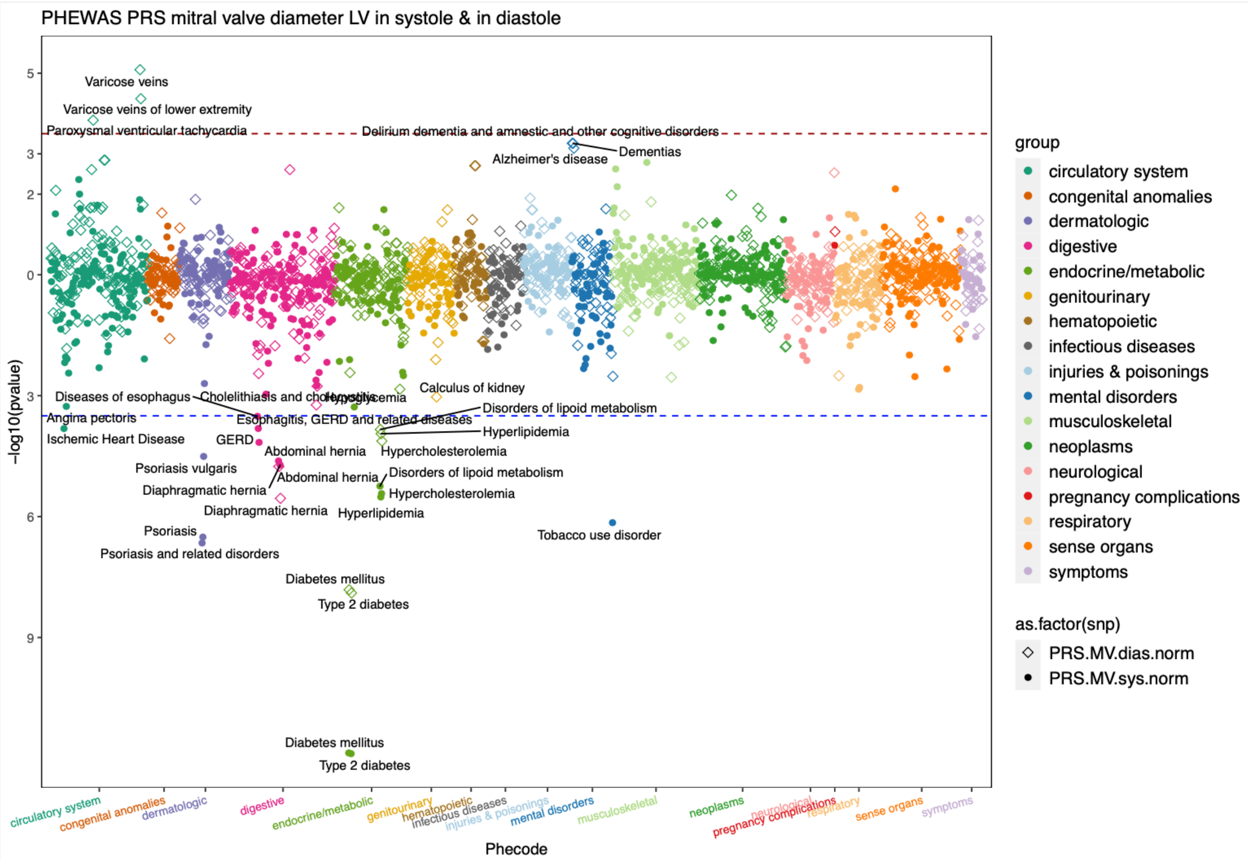

Figure S8. PheWAS of polygenic scores of mitral annulus size in systole and diastole. The absolute value of Y-axis represents the negative logarithm of the p-value with values above and below zero representing positive and negative correlations respectively. Colors represent different clinical/anatomical categories for the phecodes used. Larger PRS for annular diameter measured in diastole is associated with varicose veins paroxysmal ventricular tachycardia and negatively associated with diabetes and hypercholesterolemia. Scores were negatively associated with psoriasis and hernias affecting the cardiac surface of the diaphragm suggesting the impact of a mass effect upon cardiac anatomy.

				Aortic valve or mitral annular calcification		MV.LV.diastole		MV.LV.systole	
**SNP**	**CHR**	**POS**	**Risk Allele**	**OR**	**P-value**	**beta**	**P**	**beta**	**p**
rs10455872	6	160589086	G	2.05 [1.63, 2.57]	9.0*10-10	-0.03 [-0.19, 0.13]	0.71	-0.04 [-0.19, 0.10]	0.54
rs17659543	2	112958729	T	1.66 [1.39, 1.98]	1.5*10-8	0.08 [-0.03, 0.20]	0.16	-0.03 [-0.13, 0.08]	0.60
rs13415097	2	112959608	C	1.66 [1.39, 1.98]	1.8*10-8	0.08 [-0.03, 0.20]	0.17	-0.02 [-0.13, 0.08]	0.67

Table S6. Previous GWAS variants identified in mitral or aortic valve calcification are not detected in the present study of mitral valve annular diameter.

RS Number	Position (GRCh37)	Allele Frequencies	Haplotypes			
rs533030436	chr17:45091770	A=0.862, G=0.138	A	A	G	G
rs6504673	chr17:45096957	G=0.784, C=0.216	G	C	G	C
		**Haplotype Count**	700	167	89	50
		**Haplotype Frequency**	0.6958	0.166	0.0885	0.0497

Figure S9. Visualization of population substructure within five European populations (CEU, TSI, FIN, GBR, IBS) for two variants near *GOSR2* impacting mitral valve annular diameter. Variants display only minimal linkage R^2 0.0197, p <0.0001. Note that genome coordinates are hg19 as haplotype calculations are derived from the 1000 Genomes reference panel. (https://ldlink.nci.nih.gov/).

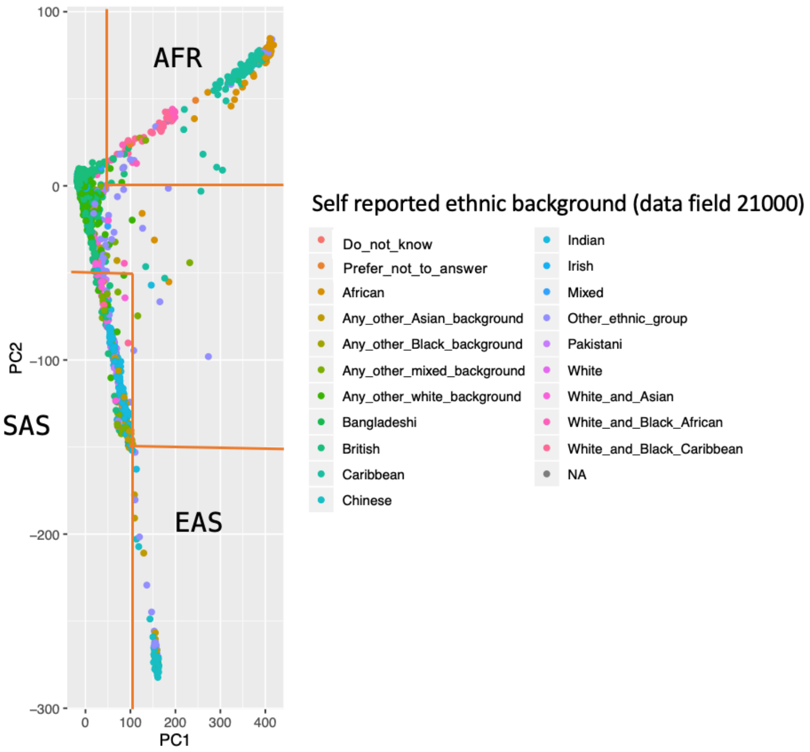

Figure S10. Comparison of self-reported ethnic background to ancestry defined by principal components analysis for the definition of discovery, replication, and population strata for trans-ethnic meta-analysis.

Table S7. Characteristics mitral valve tissue donors used for the ATAC-Seq analysis.

Sample	Sex	Age	BMI kg/m^2	Mitral Valve insufficiency (I-III)
MV1671	F	70	23.7	III
MV1700	M	65	23.8	III
MV1772	F	80	24.4	III
MV1830	F	78	24.0	III
MV1846	M	67	27.6	III
